# Quality of Life Outcomes After Endoscopic Cholesteatoma Surgery: A Prospective Cohort Study

**DOI:** 10.3390/jcm15041556

**Published:** 2026-02-16

**Authors:** Luana-Maria Gherasie, Viorel Zainea, Tamer Ebaied, Razvan Hainarosie, Corina Daniela Negrila, Andreea Rusescu, Irina-Gabriela Ionita, Catalina Voiosu

**Affiliations:** 1 General Medicine, Carol Davila University of Medicine and Pharmacy, 050474 Bucharest, Romania; luana-maria.bujor@drd.umfcd.ro (L.-M.G.);; 2 “Prof. Dr. D. Hociota” Institute of Phonoaudiology and Functional ENT Surgery, 050751 Bucharest, Romania; 3 Department of Ear, Nose and Throat, Sheikh Shakhbout Medical City (SSMC), Abu Dhabi 11001, United Arab Emirates

**Keywords:** cholesteatoma, endoscopic ear surgery, minimally invasive, COMQ-12, Glasgow Benefit Inventory, quality of life

## Abstract

**Background:** Cholesteatoma is a destructive middle ear lesion that compromises hearing and quality of life, for which endoscopic ear surgery (EES) has emerged as a minimally invasive alternative to microscopic approaches. While recurrence and audiological outcomes are frequently reported, patient-centered evaluations using validated patient-reported outcome measures (PROMs) remain limited. **Objective:** This study aimed to assess postoperative quality of life in patients undergoing exclusive endoscopic cholesteatoma surgery, using validated patient-reported outcome measures. **Methods:** We conducted a prospective observational cohort study in a tertiary referral center, enrolling 41 patients who underwent exclusive endoscopic cholesteatoma surgery over 20 months. Pre- and postoperative QoL was assessed using the Chronic Otitis Media Questionnaire-12 (COMQ-12) and the Glasgow Benefit Inventory (GBI). **Results:** COMQ-12 scores improved significantly from baseline (54.0 ± 4.2) to 12 months (10.2 ± 3.3; mean difference −43.8, 95% CI: −46.1 to −41.5; *p* < 0.001). GBI scores were consistently high, increasing from 82.6 ± 4.8 at 6 months to 84.1 ± 4.9 at 12 months (*p* < 0.001). Audiometric evaluation demonstrated a significant postoperative improvement, with the mean air–bone gap (ABG) decreasing from 52.1 ± 5.3 dB preoperatively to 26.4 ± 4.7 dB postoperatively (*p* < 0.001), indicating substantial closure of the conductive gap. **Conclusions:** Exclusive endoscopic cholesteatoma surgery yields robust QoL improvement, favourable hearing outcome, and recurrence rates similar to classic techniques at short-term evaluation.

## 1. Introduction

Cholesteatoma is a chronic, keratinizing squamous epithelial lesion of the tympanic cavity and mastoid that behaves in a locally aggressive manner, causing progressive bone erosion and potentially life-threatening complications if untreated [1]. Although rare, its destructive potential makes early diagnosis and effective surgical management imperative. Clinically, cholesteatoma may be classified into congenital, primary acquired, secondary acquired, and recurrent forms [2].

Despite the demonstrated anatomical and audiological efficacy of endoscopic ear surgery (EES), its impact on patient-perceived outcomes remains underexplored. Most available studies have focused on hearing thresholds, recurrence rates, or technical feasibility, while the effect of EES on health-related quality of life (QoL)—a significant determinant of long-term surgical success—has received limited attention.

Few studies to date have quantified QoL in cholesteatoma patients using validated PROMs, and results have been inconsistent. Therefore, a focused evaluation of QoL outcomes is warranted to determine whether the theoretical benefits of endoscopic surgery translate into meaningful patient benefits. Existing studies on quality-of-life outcomes in cholesteatoma patients are few and methodologically heterogeneous.

A summary of recent PROM-based research is presented in Table 1.

Results vary in magnitude and durability of benefit, and comparisons between surgical techniques remain underpowered.

To ensure consistency in describing cholesteatoma, we used the EAONO/JOS ChOLE classification to stage disease and focused on early-stage cholesteatomas amenable to endoscopic removal [10,11,12].

In recent decades, endoscopic ear surgery (EES) has become an increasingly accepted alternative or adjunct to conventional microscopic techniques [13]. Initially pioneered by Marchioni and colleagues, the transcanal endoscopic approach provides wide-angle and magnified visualization, enabling access to hidden recesses such as the sinus tympani, anterior epitympanum, and facial recess, where residual disease commonly persists [14]. Several comparative studies and meta-analyses have shown that EES achieves similar or lower recurrence and residual rates compared with microscopic surgery, with additional advantages including reduced surgical morbidity, shorter hospitalization, and improved cosmetic outcomes [15].

In theory, EES may offer several patient-centered advantages over traditional microscopic surgery: transcanal access avoids postauricular scars, reduces soft-tissue dissection, and may shorten recovery time, thereby improving comfort and self-perception. Furthermore, the wide-angle visualization of the middle ear recesses can enable complete disease removal through a less invasive approach, potentially minimizing residual disease and the psychological burden of repeated interventions.

However, whether these theoretical advantages translate into measurable improvements in postoperative QoL compared with conventional microscopy remains unclear. This study was therefore designed to assess postoperative QoL, hearing outcomes, and short-term disease control in patients undergoing exclusive endoscopic cholesteatoma surgery, and to compare these outcomes with those reported for the conventional microscopic approach (from the literature).

Histologically, cholesteatoma comprises three essential components: the matrix (keratinizing stratified squamous epithelium), keratin debris, and the perimatrix, a fibrovascular stroma with chronic inflammatory infiltrate [16,17,18]. The cholesteatoma perimatrix is metabolically active, promoting inflammation, epithelial proliferation, and bone resorption through cytokine release, MMP overexpression, and dysregulated apoptosis [11,12,13]. Collectively, these findings confirm that cholesteatoma is not merely a passive accumulation of keratin but an active inflammatory and hyperproliferative lesion with tumor-like biological behavior [19].

This study was designed as a prospective, analytical, monocentric investigation conducted at the “Prof. Dr. Dorin Hociota” Institute of Phonoaudiology and ENT Functional Surgery in Bucharest, Romania. Over a 20-month period, between October 2023 and May 2025, 41 patients with middle ear cholesteatoma underwent surgery using an exclusively endoscopic approach. Pre- and postoperative QoL assessments were performed using the COMQ-12 and GBI questionnaires, associated audiological, and imaging evaluations. The objective was to analyze postoperative QoL improvement, PROMs dynamics, and audiological outcomes, thereby providing an integrated, patient-centered perspective on the benefits of endoscopic cholesteatoma surgery.

**Figure 1:**
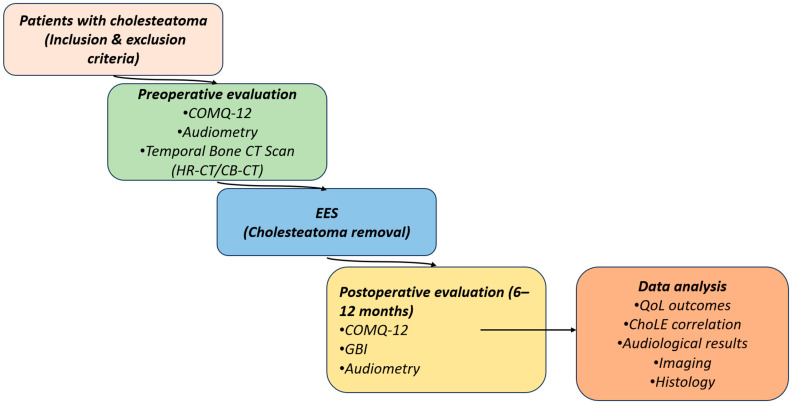
Study workflow for patients with cholesteatoma. Preoperative assessment included COMQ-12, audiometry, and temporal bone CT. Postoperative follow-up comprised COMQ-12, GBI, audiometry, and imaging at 6–12 months.

A schematic overview of patient assessment and follow-up is shown in Figure 1, and the primary and secondary objectives of the study are outlined in Table 2.

## 2. Materials and Methods

### 2.1. Study Design

This investigation was designed as a prospective observational cohort study conducted at a single tertiary referral center. The study protocol was approved by the Institutional Review Board of “Prof. Dr. Hociota” Institute of Phonoaudiology and ENT Functional Surgery (approval code: 8696) in accordance with the Declaration of Helsinki, and all participants provided written informed consent before enrollment. To minimize bias, strict inclusion and exclusion criteria were applied, excluding patients with extensive disease that would have required a microscopic or combined approach. All eligible patients scheduled for exclusive endoscopic surgery during the study period (October 2023–May 2025) were approached for enrollment. Consecutive enrollment of eligible cases further reduced the risk of selection bias. Importantly, no patients were lost to follow-up, as all 41 participants completed the planned 12-month evaluation, thereby eliminating attrition bias. The design and reporting of the study adhere to the Strengthening the Reporting of Observational Studies in Epidemiology (STROBE) guidelines [20]. The analysis encompassed both clinical outcomes and patient-reported outcomes, with pre- and postoperative quality-of-life assessments (using the COMQ-12 and GBI, detailed below) [21,22]. Eligibility further required the ability to complete the QoL questionnaires (COMQ-12 and GBI) preoperatively and postoperatively, and a planned follow-up of at least 12 months [23,24].

Exclusion criteria were defined to eliminate factors that could bias the evaluation of surgical outcomes. Patients were not eligible if the lesions extended beyond the posterior limit of the lateral semicircular canal [25]. These criteria are summarized in Table 3.

Cholesteatoma extension in each patient was documented according to the STAMCO and Chole classification system for cholesteatoma [26]. Based on Chole classification, our cohort is included in Stage 1, and all patients, based on STAMCO classification (100%, *n* = 41), had cholesteatoma involving the attic (A) and the tympanic cavity (T) [27].

**Figure 2:**
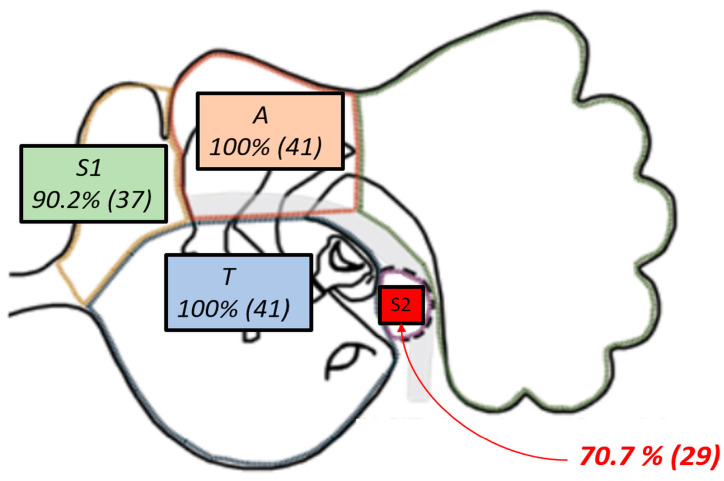
Cholesteatoma extension in the cohort: attic (A) and tympanic cavity (T) 100% (41/41); anterior difficult area (S1) 90.2% (37/41); posterior difficult area (S2) 70.7% (29/41).

Additionally, 90.2% of cases showed extension into the anterior epitympanic recess (S1, also known as the supratubal recess or “anterior difficult area”), and 70.7% had disease involving the posterior tympanic recess (S2, the sinus tympani or “posterior difficult area”), none of the included cholesteatomas extended beyond the posterior limit of the lateral semicircular canal [28]. The distribution of cholesteatoma involvement in the study cohort is illustrated in Figure 2.

No a priori sample size calculation was performed for this observational study. Instead, we included all consecutive eligible patients over the 20-month period (October 2023–May 2025), given the exploratory nature of the study and the relatively limited pool of exclusively endoscopic cases. At the preoperative consultation, all patients with suspected cholesteatoma underwent high-resolution temporal bone CT to assess disease extension and determine eligibility for an exclusively endoscopic approach. None of these patients required intraoperative conversion from an endoscopic to a combined or microscopic approach.

We included patients with middle ear cholesteatoma that was judged (based on preoperative CT and endoscopic evaluation) to be completely resectable via a transcanal endoscopic approach. Patients requiring a mastoidectomy or combined approach (due to extensive mastoid disease or other factors) were not included in this series. During the study period, 451 cholesteatoma surgeries-canal wall-up (CWU) and canal wall-down (CWD) mastoidectomies, were performed via conventional microscopy at our center; our endoscopic series thus represents a small, select subset of cases.

### 2.2. Surgical Technique

**Figure 3:**
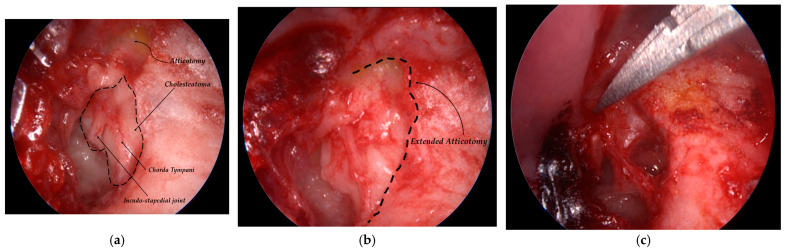
Intraoperative endoscopic views of middle ear cholesteatoma surgery. (**a**) Cholesteatoma with displacement of the chorda tympani and involvement of the incudo-stapedial joint. (**b**) Extended atticotomy to improve exposure and facilitate complete disease removal. (**c**) Intraoperative view during dissection.

All procedures were performed under general anesthesia via an exclusive endoscopic transcanal approach. A rigid Hopkins rod-lens endoscope (0° or 30°, 3 mm diameter, 14 cm length), integrated with a Full-HD Olympus camera system, was used for intraoperative visualization [29]. Angled endoscopes were employed as needed to visualize and ablate cholesteatoma from hidden recesses, including the sinus tympani, anterior epitympanic (supratubal) recess, and facial recess [30,31]. After complete disease removal, tympanic membrane reconstruction was performed using tragal cartilage graft, and if ossicular chain reconstruction was required, a partial or total ossicular replacement titanium prosthesis (PORP or TORP) was placed to restore continuity of the ossicular chain [32]. Representative intraoperative endoscopic findings are illustrated in Figure 3.

In most cases, radiologic and intraoperative findings were concordant. However, in three patients, the surgical plan was modified due to discordant findings and anesthetic considerations. These patients presented with significant medical comorbidities that temporarily contraindicated general anesthesia, leading to a delay of approximately six months between imaging and surgery [33]. Preoperative imaging revealed an approximately 1 cm tegmen tympani defect; therefore, a combined surgical approach was planned to allow safe intraoperative assessment and potential repair of a CSF fistula. Although no fistula was identified, these cases were excluded because a mixed endoscopic–microscopic approach was used.

Intraoperative bleeding was assessed qualitatively by the operating surgeon and documented when it resulted in prolonged surgical time or temporary impairment of the endoscopic visual field. Prolonged operative time related to bleeding was defined as an increase of up to 20 min required to achieve adequate hemostasis and restore a stable endoscopic field. Bleeding management strategies included the use of suction-integrated instruments, vasoconstrictor-soaked pledgets, meticulous atraumatic tissue handling, effective local anesthesia, and selective administration of tranexamic acid when indicated.

Intraoperative bleeding led to a modest prolongation of surgical time in 4 out of 41 cases (9.8%). One patient was receiving antiplatelet therapy, while three patients presented with marked inflammatory changes of the middle ear mucosa. In these cases, the operative time was prolonged by no more than 20 min to allow adequate hemostasis and stabilization of the endoscopic visual field. No procedure required conversion to a microscopic or combined approach due to bleeding. Adequate bleeding control was achieved in all cases, allowing completion of the surgery via an exclusive endoscopic approach.

### 2.3. Clinical and Audiological Assessment

Preoperative and postoperative hearing was evaluated with standard pure-tone audiometry. Air-conduction and bone-conduction thresholds were measured at 0.5, 1, 2, and 4 kHz, and the four-frequency pure-tone average (PTA) was calculated for each ear. Postoperative audiometric testing was performed at approximately 3, 6, and 12 months [8].

### 2.4. Imaging Assessment

All patients underwent high-resolution computed tomography (CT) of the temporal bone preoperatively to delineate the extent of disease and identify any pertinent anatomical variations, focusing on the Fallopian canal and stapes-associated lesions [34]. For postoperative surveillance, a non-echo-planar diffusion-weighted MRI (non-EPI DWI MRI) was obtained at 12 months after surgery to detect the residual or recurrent cholesteatoma [35].

### 2.5. Quality of Life Assessment

Quality of life was assessed using two validated patient-reported outcome measures. The COMQ-12, a 12-item disease-specific instrument, was completed preoperatively and again at 6 and 12 months postoperatively [3]. This questionnaire yields a total score from 0 to 60, with higher scores indicating a greater symptom burden and impact on daily life. The GBI was administered at 6 months and 12 months after surgery to evaluate the patient’s perceived benefit from the intervention [3]. The GBI is a generalized postoperative questionnaire that produces an overall score ranging from −100 (maximum negative impact) to +100 (maximum positive benefit), with 0 indicating no change; it encompasses subscales for general, social, and physical health domains [36].

### 2.6. Statistical Analysis

Data analysis was performed using Python (version 3.11.6) (with the pandas—version 2.1.1 and SciPy libraries—version 1.11.3). Continuous variables are presented as mean ± standard deviation (SD) if they were approximately normally distributed, or as median with interquartile range (IQR) if they exhibited a skewed distribution. The normality of data (and of paired differences in pre/post comparisons) was evaluated with the Shapiro–Wilk test.

For paired pre- vs. post-operative comparisons within the same patients, appropriate paired statistical tests were applied. If the distribution of the paired differences met the assumption of normality, a paired Student’s *t*-test was used; otherwise, the non-parametric Wilcoxon signed-rank test was employed. This approach was used to evaluate changes in COMQ-12 scores from baseline to 6 and 12 months, to compare GBI scores between 6 months and 12 months post-surgery, and to assess improvements in PTA between the preoperative and postoperative audiograms. Associations between continuous variables (for example, the correlation between improvement in COMQ-12 score and gain in PTA, or between 12-month GBI score and patient age or hearing improvement) were examined using Pearson’s correlation coefficient for approximately normally distributed data, or Spearman’s rank-order correlation for non-normal data. Comparisons across more than two independent groups (e.g., comparing outcomes among different ossiculoplasty materials or techniques) were performed using one-way analysis of variance (ANOVA) if the data were normal, or the Kruskal–Wallis test if normality assumptions were not met. Relationships between categorical variables (such as the presence of MRI-confirmed residual/recurrent cholesteatoma vs. the type of tympanoplasty or ossiculoplasty performed) were analyzed with the chi-square test, or with Fisher’s exact test for 2 × 2 tables when expected cell counts were low. All hypothesis tests were two-tailed, and a *p*-value < 0.05 was considered statistically significant.

## 3. Results

### 3.1. Patient Characteristics

A total of 41 patients underwent exclusive endoscopic cholesteatoma surgery and were included in the analysis. The mean age at surgery was 41.0 ± 14.4 years (range 19–65). There was a predominance of female patients (25 females, 61.0%), and the right ear was affected slightly more often than the left (23 right ears, 56.1%). According to the CES classification [37], 16 cases (39.0%) were classified as CES0, 13 (31.7%) as CES 1p, and 12 (29.3%) as CES 1a. In terms of disease staging by the ChOLE system, 21 patients (51.2%) had Stage I disease and 20 (48.8%) had Stage II. Ossicular chain reconstruction was required in most patients (70.7%): 11 received a cartilage graft, 10 a partial ossicular replacement prosthesis (PORP), and eight a total ossicular replacement prosthesis (TORP), while 12 patients (29.3%) did not require ossiculoplasty.

All patients completed at least 12 months of postoperative follow-up. The demographic and clinical characteristics of the cohort are summarized in Table 4.

### 3.2. Quality of Life Outcomes

**Figure 4:**
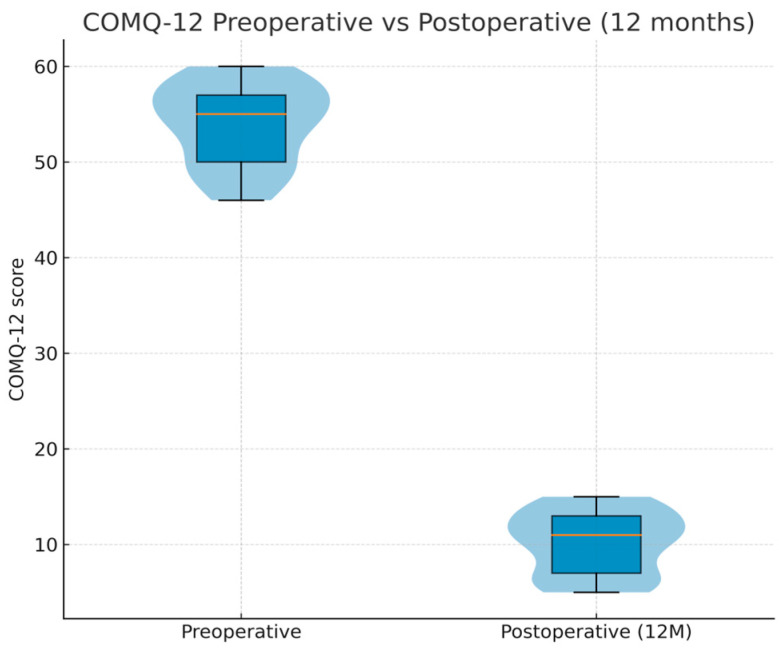
COMQ-12 scores before and after surgery. Violin plots with boxplot overlay comparing COMQ-12 total scores preoperatively and 12 months postoperatively. A marked reduction in symptom burden and disease impact was observed (paired *t*-test, *p* < 0.001).

Preoperative COMQ-12 scores were high, indicating considerable symptom burden and disease-related impairment (mean 54.0 ± 4.2 out of 60). Postoperatively, COMQ-12 scores improved at all assessed intervals. At 3 months, the mean COMQ-12 had decreased to 41.9 ± 7.2 (paired *t*-test vs. baseline, *p* < 0.001). Further improvement was observed by 6 months (38.0 ± 5.3, *p* < 0.001 vs. baseline) and by 12 months (10.2 ± 3.3, *p* < 0.001 vs. baseline). This corresponds to an average reduction of approximately ~44 points (≈81% improvement) in COMQ-12 scores from the preoperative baseline to 12 months, corresponding to an average decrease of roughly 44 points (≈81% improvement; 95% CI: −46.1 to −41.5). Figure 4 illustrates the distribution of COMQ-12 scores preoperatively and at 12 months, and detailed longitudinal results are presented in Table 5.

Values are mean ± SD. Paired *t*-tests were used to compare each postoperative time point to the preoperative baseline; all postoperative scores were significantly improved compared to baseline (*p* < 0.001 for each comparison).

Patient-reported health benefit, as measured by the GBI, was positive at both postoperative time points and increased over time. At 6 months, the mean GBI score was 82.6 ± 4.8, indicating a high level of self-reported benefit. By 12 months, the mean GBI had further increased to 84.1 ± 4.9, a modest but statistically significant rise (mean difference 1.5, 95% CI: 0.8 to 2.2; *p* < 0.001; paired *t*-test comparing 12 vs. 6 months, *p* < 0.001). These findings indicate that patients experienced substantial and sustained improvement in health-related quality of life, with a slight additional gain between 6 and 12 months. The GBI outcomes are summarized in Table 6.

### 3.3. Audiological Results

Audiometric evaluation demonstrated postoperative improvement in hearing. The mean preoperative four-frequency pure-tone average air-conduction threshold was 52.1 ± 5.3 dB HL, which improved to 26.4 ± 4.7 dB HL at 12 months after surgery (paired *t*-test, *p* < 0.001). This improvement corresponds to an average hearing gain of approximately 25.7 dB (95% CI: 23.9 to 27.5), reflecting a substantial closure of the air-bone gap (mean hearing gain −25.7 dB; 95% CI: −27.5 to −23.9). No major complications were encountered in our cohort. In particular, there were no cases of sensorineural hearing loss, facial nerve palsy, persistent taste disturbance, or other significant postoperative complications.

### 3.4. Recurrence and Residual Disease

At 12 months postoperatively, all patients underwent non–EPI DWI MRI as part of the follow-up protocol, with no residual or recurrent cholesteatoma detected. Further surveillance will be conducted in accordance with established guidelines and recommendations to identify potential recurrence.

### 3.5. Correlations and Subgroup Analyses

**Figure 5:**
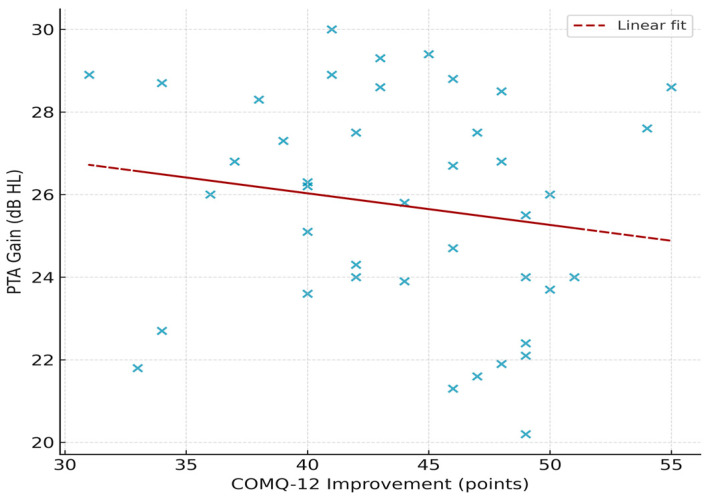
Correlation between COMQ-12 improvement and hearing gain. Scatter plot illustrating the relationship between postoperative improvement in COMQ-12 scores (preoperative–12 months) and pure-tone average (PTA) gain. Each dot represents an individual patient (*n* = 41). The dashed line indicates the linear regression fit. No significant correlation was observed (Pearson r = −0.16, *p* = 0.31).

Correlation analyses did not show any significant relationships between hearing outcomes and patient-reported outcome measures. In particular, there was no significant correlation between the magnitude of COMQ-12 improvement (preoperative minus 12-month score) and the hearing gain in dB (Pearson *r* = −0.16, *p* = 0.31). Similarly, 12-month GBI scores showed no significant association with hearing gain (Spearman ρ = 0.11, *p* = 0.50) or with patient age (Spearman ρ = −0.08, *p* = 0.63). These findings suggest that improvements in hearing and quality of life were largely independent of each other and were not influenced by patient age. The scatter plot in Figure 5 illustrates the lack of correlation between COMQ-12 improvement and hearing gain, and the correlation results for all variables are detailed in Table 7.

Subgroup analyses similarly revealed no significant differences in outcomes based on surgical subgroup or disease stage. Patients were stratified by type of ossiculoplasty performed (none, cartilage graft, PORP, TORP), but no significant differences were found among these groups in mean COMQ-12 improvement or in 12-month GBI scores (one-way ANOVA for both comparisons; *p* > 0.4 in each case). There was also no association between the type of ossicular reconstruction and the incidence of cholesteatoma recurrence on follow-up MRI (chi-square test, *p* = 0.65). Furthermore, outcome measures did not differ significantly by disease extent: patients with cholesteatomas not involving the anterior epitympanic recess (S1) and the sinus tympani (S2) had postoperative COMQ-12 and GBI results comparable to those of patients with cholesteatomas extending into these areas (no significant between-group differences, *p* > 0.5).

We found no significant differences in outcomes based on disease extent or reconstruction type. Patients whose cholesteatomas extended into the anterior epitympanic recess or sinus tympani had postoperative COMQ-12 and GBI improvements comparable to those without such extension (*p* > 0.5 for all comparisons). Similarly, the degree of hearing improvement and QoL benefit did not significantly vary among different ossiculoplasty methods (cartilage vs. PORP vs. TORP, *p* > 0.5).

## 4. Discussion

### 4.1. Interpretation of Findings

The combined approach is indeed valuable in complex or extended cases, where endoscopic inspection after microscopic removal may help confirm complete disease clearance. In clinical practice, both instruments are complementary rather than competitive, and their combined use likely represents the optimal balance between visualization and surgical control. The findings of this study should therefore be interpreted in the context of surgical strategy. While endoscopic techniques expand the limits of minimally invasive otology, optimal cholesteatoma management often depends on individualized decisions, the surgeon’s experience, and the integration of both visualization modalities when appropriate.

Bleeding control is a recognized limitation of endoscopic ear surgery, given the one-handed nature of the technique and the absence of continuous bimanual suction available in microscopic surgery. In our cohort, intraoperative bleeding resulted in a modest prolongation of surgical time in a small subset of patients (9.8%), primarily in those receiving antiplatelet therapy or presenting with significant inflammatory changes of the middle ear mucosa. Nevertheless, bleeding was effectively managed in all cases without the need for conversion to microscopic surgery. This was facilitated by the use of suction-integrated instruments, effective local anesthesia, careful atraumatic handling of inflamed tissues, and selective administration of tranexamic acid when necessary. These measures allowed restoration of a stable endoscopic field within a limited additional operative time (≤20 min). Our findings suggest that, in appropriately selected cases and in experienced hands, intraoperative bleeding during exclusive endoscopic cholesteatoma surgery can be adequately controlled, although extensive disease or uncontrolled hemorrhage may still favor a microscopic or combined approach.

The present prospective cohort study demonstrates that exclusive endoscopic management of middle ear cholesteatoma is associated with significant improvements in patient-reported quality of life. COMQ-12 scores decreased markedly at all postoperative intervals, reflecting both symptom relief and reduced disease impact. Meanwhile, GBI scores confirmed a sustained perception of benefit, with further improvements observed between the 6- and 12-month follow-ups. In practical terms, patients experienced a marked improvement in disease burden, with COMQ-12 scores dropping by about 44 points (95% CI: −46.1 to −41.5). At the same time, they reported a sustained benefit in quality of life, as reflected by a 1.5-point increase in GBI (95% CI: 0.8 to 2.2). Importantly, hearing function also improved, with a mean gain of 25.7 dB (95% CI: −27.5 to −23.9), indicating a substantial closure of the air–bone gap [38,39].

It is noteworthy that our patients’ baseline COMQ-12 scores were near the upper limit of the scale. This raises the possibility of a ceiling effect—when starting at such a high score, even modest clinical improvements can yield a large drop in the score. Likewise, by 12 months, many patients had scores approaching the minimum, suggesting a potential floor effect for post-operative assessment. These instrument limitations mean that, while a ~44-point improvement is impressive, part of this change may be due to starting from an extreme baseline. Additionally, patients with extremely severe symptoms (and thus high COMQ-12) might have been more likely to undergo surgery, which is another facet of selection bias. We acknowledge these factors as they may somewhat exaggerate the perceived magnitude of QoL improvement.

Our findings align with a growing body of evidence highlighting the advantages of endoscopic ear surgery over traditional microscopic approaches [40,41]. Several systematic reviews and meta-analyses have shown that cholesteatoma recurrence and residual disease rates are lower with endoscopic techniques [42]. This is largely attributed to the endoscope’s wide-angle visualization, which allows inspection of hidden recesses such as the sinus tympani and anterior epitympanum [43]. For example, Li et al. (2021) analyzed 13 studies and found that the recurrence risk was nearly halved with endoscopic surgery (RR ≈ 0.51), and residual cholesteatomas were significantly less frequent (RR ≈ 0.68) compared to microscopic surgery [44]. Similarly, a systematic review by Nair et al. (2022) confirmed lower recurrence rates with exclusively endoscopic techniques [9]. These benefits extend to pediatric populations as well. Basonbul et al. (2021) reported that using endoscopes for cholesteatoma removal halved the residual disease rate (RR = 0.48, *p* < 0.001) compared to conventional methods [45].

Regarding postoperative surveillance, a second-look procedure remains the traditional gold standard for detecting residual or recurrent cholesteatoma, offering direct inspection and immediate removal if necessary. However, it carries the inherent risks and morbidity of a repeat operation. In recent years, non-EPI DWI-MRI has proven highly sensitive and specific for detecting residual disease ≥ 3 mm, providing a reliable, noninvasive alternative in canal-wall-up and endoscopic cases. In our institution, non-EPI DWI-MRI was preferred over second-look surgery as a standard follow-up tool, particularly in patients with favorable intraoperative visualization and complete disease removal. Nevertheless, second-look surgery remains advisable in selected cases, especially in children or when intraoperative exposure is limited. While encouraging, longer-term follow-up is required, since late recurrences beyond the first postoperative year are well-documented in the literature. However, the 12-month follow-up period remains relatively short, and late recurrences are well documented in the literature. Therefore, our findings should be interpreted with caution, and longer-term surveillance is warranted to confirm the durability of disease control. Evidence from randomized controlled trials (RCT) also supports these findings. In a recent RCT, Hamel et al. (2023) showed that endoscopically treated patients had significantly lower recurrence (7.5% vs. 27.5%) and residual disease rates (5.0% vs. 22.5%) compared to those who underwent conventional microscopic tympanoplasty [46]. Importantly, these advantages were achieved without compromising surgical access or increasing morbidity, confirming that enhanced visualization of hidden areas can translate into clinically relevant benefits [47].

With respect to hearing outcomes, most studies report no significant differences between endoscopic and microscopic approaches. Meta-analyses have consistently found that both techniques achieve comparable postoperative air–bone gap closures and overall audiological outcomes [9,48]. While some series suggest that the endoscopic approach can facilitate ossicular preservation due to better visualization around the ossicular chain, the overall magnitude of hearing gain has not differed significantly between techniques [49].

In our cohort, hearing outcomes improved, yet this improvement was not directly mirrored by changes in PROM scores, reinforcing the notion that QoL and audiological gains may evolve independently.

Preoperative COMQ-12 scores were very high (mean 54.0 out of 60, with many patients scoring above 50), indicating a considerable symptom burden and impact on daily life prior to surgery. Patient-reported outcomes are an increasingly important measure of surgical success. Raemy et al. (2025) compared postoperative QoL in patients undergoing endoscopic versus microscopic cholesteatoma surgery and observed higher QoL scores in the endoscopic cohort, although the difference was not statistically significant-likely due to limited sample size or case selection [4]. Our findings reinforce this trend by demonstrating a significant, sustained improvement in QoL after exclusive endoscopic surgery. In addition to QoL gains, prior studies have noted other advantages of endoscopic techniques, including less postoperative pain, faster wound healing, and better cosmetic outcomes. For example, Hamel et al. reported that tympanic membrane grafts healed about two weeks faster in the endoscopic group compared to the microscopic cohort in their randomized trial [46].

Regarding safety and complications, both approaches are generally considered safe, with low rates of major adverse events [50,51]. Indeed, comparative series report no significant differences in overall complication rates between endoscopic and microscopic surgery. However, certain specific morbidities may be less frequent with endoscopy. For example, Otsuka et al. (2024) reported that taste disturbance from chorda tympani injury occurred in 11% of microscopic cases but in 0% of endoscopic cases—a statistically significant disparity [52]. Similarly, wound infections and postoperative balance disturbances have been reported less often with endoscopic procedures, although these differences do not always reach statistical significance [53].

Taken together, evidence from the past decade suggests that endoscopic cholesteatoma surgery yields outcomes at least comparable to—and in some respects superior to—those of traditional microscopic techniques [54,55]. However, since our study lacked a direct control group, we base these comparisons on literature rather than on internal data. Nevertheless, it should be emphasized that most endoscopic series have involved relatively limited disease; in cases of extensive mastoid involvement, the microscope remains indispensable [56]. An individualized approach tailoring the surgical technique to the disease extent and anatomic conditions, therefore, remains essential [57].

### 4.2. Limitations

This study has several limitations that should be acknowledged. First, the relatively small sample size (*n* = 41) and single-center design may limit the generalizability of the findings. It was not powered by a formal a priori sample size calculation, and the final cohort size was determined by the number of consecutive eligible patients treated during the study period. Consequently, the study may not have been sufficiently powered to detect small but potentially clinically relevant differences, particularly in subgroup or secondary analyses. However, the study was designed to reflect real-world clinical practice, and the inclusion of a consecutive patient series reduces selection bias and enhances the external validity of the findings. Within this context, the observed outcomes provide meaningful clinical insights into endoscopic surgical management, although future prospective and adequately powered multicenter studies are needed to further validate these results. Second, the follow-up period was limited to 12 months. This underscores the need for extended follow-up to assess the long-term efficacy of exclusive endoscopic cholesteatoma surgery fully.

Third, our inclusion of only those patients amenable to an exclusively endoscopic approach introduces a selection bias. Patients with more extensive disease (e.g., mastoid involvement beyond endoscopic access or advanced stages) were treated with microscopic or combined techniques and thus not included. As a result, our cohort likely had inherently more favorable disease characteristics (limited stage and extent), which could in part explain the excellent outcomes. This limits generalizability; the results may not translate to unselected cholesteatoma cases or those requiring traditional open surgery.

We intentionally limited our study to an exclusively endoscopic cohort to assess the outcomes of this technique on its own merits. Although surgeons adopt a hybrid endoscope-microscope approach for complex cases, here we wanted to characterize the results attainable by endoscope alone, without the confounding effects of mixed techniques

Finally, the absence of a control group treated with conventional microscopic surgery precludes direct comparison; therefore, we relied on data from the existing literature for contextualization. Despite these limitations, the study provides valuable prospective evidence that exclusive endoscopic surgery can achieve substantial improvements in quality of life and hearing, with a very low recurrence rate in appropriately selected cases.

Although preoperative CT remains essential for planning, intraoperative findings may occasionally diverge, particularly when imaging is dated or comorbidities delay surgery. In our experience, three such cases required intraoperative conversion from exclusive endoscopic to combined CWU microscopic mastoidectomy to ensure complete inspection of the mastoid air cells.

## 5. Conclusions

This prospective study demonstrates that exclusive endoscopic cholesteatoma surgery can achieve significant improvements in both patient-reported and clinical outcomes [55]. Postoperatively, patients experienced marked quality-of-life gains—evidenced by improved COMQ-12 and GBI scores—alongside meaningful hearing improvements. Notably, we found no correlation between the extent of hearing gain and QoL improvement, suggesting that audiological success does not automatically translate into better patient-perceived outcomes; this highlights the importance of directly measuring patient-reported benefits in chronic ear disease [58,59]. Furthermore, the positive outcomes in our cohort were consistent across different surgical scenarios: we observed no significant variation in results based on the ossiculoplasty type or cholesteatoma stage. This suggests that the advantages of the endoscopic approach are broadly applicable, with effective disease control and functional recovery achievable even in the more extensive cases within our cohort [60,61].

Collectively, these findings underscore that an exclusively endoscopic approach to cholesteatoma is a safe, feasible, and patient-centered surgical strategy. By avoiding external incisions and providing wide-angle visualization of the middle ear, this minimally invasive technique enables thorough cholesteatoma eradication while minimizing morbidity, aligning with prior reports of endoscopic ear surgery as a “safe and effective transcanal alternative” to conventional postauricular procedures [62]. Looking ahead, longer-term follow-up is warranted to assess the durability of cholesteatoma control and hearing outcomes after endoscopic surgery, and multicenter studies would help validate these results across diverse populations [63]. Future controlled studies could directly compare QoL outcomes between techniques, although ethical/practical constraints may limit randomization.

In conclusion, exclusive endoscopic ear surgery is a safe and effective technique for selected cases of limited cholesteatoma, providing outcomes comparable to microscopic surgery in terms of hearing and patient-reported quality of life, while offering the advantages of a minimally invasive, scarless approach. For extensive disease involving the mastoid or labyrinthine structures, the microscope—alone or in combination with endoscopic assistance—remains indispensable. Also, the microscope remains indispensable in extensive disease or situations requiring advanced bleeding control.

Future multicenter studies with longer follow-up are warranted to validate these findings and to further define the optimal integration of endoscopic and microscopic techniques in cholesteatoma management.

## Figures and Tables

**Table 1:** Overview of published studies assessing quality-of-life outcomes in cholesteatoma using validated PROMs. While disease-specific instruments like COMQ-12, CES, or ZCMEI-21 are commonly used, their application remains limited in cholesteatoma-specific cohorts.

Theme	Key Findings	Representative Studies
**Use of PROMs in** **Cholesteatoma** **Surgery**	Phillips et al. (2021) [3] validated the COMQ-12 across multiple populations and languages, demonstrating that it captures hearing disability and its impact. Raemy et al. (2024) reported greater QoL gains with endoscopic vs. microscopic surgery, though the difference was not statistically significant.	Phillips et al. (2021) [3] Raemy et al. (2024) [4]
**QoL Outcomes**	Most studies using COMQ-12, CES, or ZCMEI-21 report significant improvement in disease-specific QoL after cholesteatoma surgery. However, the magnitude of improvement and long-term stability vary. For instance, Baetens et al. (2019) showed near-normal COMQ-12 scores post CWU-BOT, while Quaranta et al. (2014) found similar QoL between CWD and CWU patients. Postoperative hearing was a major determinant of QoL in many cohorts.	Baetens et al. (2019) [5] Quaranta et al. (2014) [6]
**QoL vs. traditional measured outcomes**	Studies show that patient-reported QoL does not always correlate with clinical outcomes such as hearing gain or recurrence. Weiss et al. (2020) and Bächinger et al. (2020) found that better postoperative hearing correlated with QoL, but residual symptoms, emotional and social aspects also influenced scores.	Weiss et al. (2020) [7] Bächinger et al. (2020) [8]
**Endoscopic Surgery and PROMs**	Endoscopic cholesteatoma surgery has been associated with lower recurrence rates and comparable hearing outcomes, but data on PROMs remain scarce. Nair et al. (2021) and Raemy et al. (2025) found improved symptom-specific QoL post-EES, though general QoL (SF-12, GBI) did not always reflect significant change. Further PROM-based trials are needed to validate patient-centered benefits.	Nair et al. (2021) [9], Raemy et al. (2024) [4]

**Table 2:** Study objectives: to evaluate quality of life outcomes after endoscopic cholesteatoma surgery.

Primary Objective	To Assess the Impact of Endoscopic Cholesteatoma Surgery on Health-Related Quality of Life (QoL) Using Two Validated PROMs: COMQ-12 and GBI.
**Secondary** **objectives**	To compare pre- and postoperative COMQ-12 scores and quantify improvement in specific domains (symptoms, psychosocial impact, health service use). To evaluate postoperative GBI scores (general, social, and physical benefit). To analyze correlations between PROMs and clinical variables: audiological outcomes (PTA gain) and disease classification (STAMCO)

**Table 3:** Inclusion and exclusion criteria for patient selection in the study evaluating quality-of-life outcomes after endoscopic cholesteatoma surgery.

Inclusion Criteria	Exclusion Criteria
Diagnosis of primary or recurrent cholesteatoma (confirmed by clinical exam and imaging)	Extensive disease requiring a retroauricular approach
Scheduled for exclusive endoscopic cholesteatoma surgery	Contraindication to general anesthesia
Ability to complete pre- and postoperative QoL questionnaires (COMQ-12 and GBI)	Cognitive impairment or inability to complete questionnaires
Minimum planned follow-up of ≥12 months	
Written informed consent obtained	

**Table 4:** Demographic and clinical characteristics of the study cohort. Values are expressed as mean ± SD (range) for continuous variables and as counts with percentages for categorical variables. All patients underwent exclusive EES with a minimum follow-up of 12 months.

Characteristic	Value
Age (mean ± SD, range)	41.0 ± 14.4 (19–65)
Sex (M/F, %)	M: 16 (39.0%), F: 25 (61.0%)
Operated side (Right/Left, %)	Right: 23 (56.1%), Left: 18 (43.9%)
Chole classification	Stage 1: 41 (100%)
STAMCO classification	Attic (A) and tympanic cavity (T) 100% (41) anterior difficult area (S1) 90.2% (37) posterior difficult area (S2) 70.7% (29)
Type of intervention	Exclusive EES: 41 (100%)
Type of reconstruction	Cartilage tympanoplasty: 12 (29.3%); Incus Interposition: 11 (26.8%); PORP: 10 (24.4%); TORP: 8 (19.5%)
Follow-up (mean, range)	12 months (all patients)

**Table 5:** COMQ-12 scores preoperatively and at 3, 6, and 12 months postoperatively.

Timepoint	COMQ-12 Score (Mean ± SD)	*p*-Value (vs. Preop)
Preoperative	54.0 ± 4.2	
3 months	41.9 ± 7.2	<0.001
6 months	38.0 ± 5.3	<0.001
12 months	10.2 ± 3.3	<0.001

**Table 6:** Glasgow Benefit Inventory (GBI) scores at 6 and 12 months postoperatively. Values are mean ± SD. A paired *t*-test was used to compare 12-month and 6-month scores; there was a significant increase in GBI between 6 and 12 months.

Timepoint	GBI Score (Mean ± SD)	*p*-Value (12 vs. 6 Months)
6 months	82.6 ± 4.8	–
12 months	84.1 ± 4.9	<0.001

**Table 7:** Correlations between patient-reported outcome measures (PROMs), audiological outcomes, and patient variables. Pearson or Spearman correlation coefficients (r/ρ) are shown as appropriate (Pearson used for normally distributed differences, Spearman for ordinal/non-normal data). *n* = 41 for all analyses. No statistically significant correlations were observed. (PTA = pure-tone average hearing level gain in dB; COMQ-12 improvement = decrease in COMQ-12 score from preop to 12 months).

Variable 1	Variable 2	*n*	Method	Correlation (r/ρ)	*p*-Value
COMQ-12 improvement	PTA gain (dB)	41	Pearson	–0.16	0.31
GBI (12 months)	PTA gain (dB)	41	Spearman	0.11	0.50
GBI (12 months)	Age (years)	41	Spearman	–0.08	0.63
COMQ-12 score (12 months)	Age (years)	41	Spearman	0.17	0.30

## Data Availability

The data presented in this study are openly available in FigShare at https://figshare.com/s/d1fb6bff3bda875b812b (accessed on 11 February 2026).

## Abbreviations

The following abbreviations are used in this manuscript:

ABG	Air–Bone Gap
ANOVA	Analysis of Variance
Bax	Bcl-2-associated X protein
Bcl-2	B-cell lymphoma 2
CES	Endoscopic classification of the external auditory canal
ChOLE	Cholesteatoma extension, Ossicular chain status, Life-threatening complications, and Eustachian tube/mastoid status
COMQ-12	Chronic Otitis Media Questionnaire-12
CT	Computed Tomography
DWI-MRI	Diffusion-Weighted Magnetic Resonance Imaging
EAONO	European Academy of Otology and Neuro-Otology
EES	Endoscopic Ear Surgery
EGF	Epidermal Growth Factor
GBI	Glasgow Benefit Inventory
IHC	Immunohistochemistry
IL	Interleukin
IQR	Interquartile Range
JOS	Japanese Otological Society
LMP	Low Molecular Mass Polypeptide
MMP	Matrix Metalloproteinase
MRI	Magnetic Resonance Imaging
OPG	Osteoprotegerin
PCNA	Proliferating Cell Nuclear Antigen
PROMs	Patient-Reported Outcome Measures
PTA	Pure-Tone Average
QoL	Quality of Life
RANKL	Receptor Activator of Nuclear Factor Kappa-Β Ligand
RCT	Randomized Controlled Trial
SD	Standard Deviation
STAMCO	S—difficult visualisation areas, TAMCO-Tympanic cavity, Attic, Mastoid, Complications, and Ossicular chain involvement
TGF-β	Transforming Growth Factor-beta
TNF-α	Tumor Necrosis Factor-alpha
